# Iron Absorption: Molecular and Pathophysiological Aspects

**DOI:** 10.3390/metabo14040228

**Published:** 2024-04-17

**Authors:** Margherita Correnti, Elena Gammella, Gaetano Cairo, Stefania Recalcati

**Affiliations:** Department of Biomedical Sciences for Health, University of Milan, 20133 Milan, Italy; margherita.correnti@unimi.it (M.C.); elena.gammella@unimi.it (E.G.); stefania.recalcati@unimi.it (S.R.)

**Keywords:** iron, intestine, absorption, regulation, iron homeostasis, gut microbiota

## Abstract

Iron is an essential nutrient for growth among all branches of life, but while iron is among the most common elements, bioavailable iron is a relatively scarce nutrient. Since iron is fundamental for several biological processes, iron deficiency can be deleterious. On the other hand, excess iron may lead to cell and tissue damage. Consequently, iron balance is strictly regulated. As iron excretion is not physiologically controlled, systemic iron homeostasis is maintained at the level of absorption, which is mainly influenced by the amount of iron stores and the level of erythropoietic activity, the major iron consumer. Here, we outline recent advances that increased our understanding of the molecular aspects of iron absorption. Moreover, we examine the impact of these recent insights on dietary strategies for maintaining iron balance.

## 1. Introduction

Iron, the most abundant element on earth [1], in the context of the competition for its possession between mammalian hosts and bacterial pathogens is a driver of evolution [2]. In fact, iron is an essential microelement for the fundamental biological processes of living organisms; only two lifeforms not requiring iron are known (*Borrelia burgdorferi* and *Lactobacilli*) [3]. Indeed, the role of iron availability on bacterial life and virulence is so important that an iron-rich environment can re-establish the threat of harmless microorganisms, as shown by the peculiar case of a scientist unknowingly suffering from hereditary hemochromatosis (HH), a genetic disease characterized by iron overload, who died of septicemic plague after being exposed to a weakened form of *Yersinia pestis* [4].

Both iron scarcity and excess may be deleterious; indeed, iron deficiency, which affects more than a billion people [5], causes anemia and impairs growth, but can protect against some bacterial infections and malaria [6]. On the other hand, insufficient iron availability impairs immunity; therefore, the “correct” amount of iron is required for health.

Iron participates as a cofactor in several highly conserved biological processes; about 400 proteins are numbered in the iron proteome [7]. In mammals, iron is involved in oxygen transport and storage by heme in hemoglobin and myoglobin, respectively, but also in energy metabolism as a cofactor for the heme and iron-sulfur proteins necessary for mitochondrial function (e.g., Krebs cycle, and electrons transport chain). Moreover, as a cofactor for ribonucleotide reductase, iron is required for DNA synthesis and cell replication [8]. Interestingly, a recent study showed that iron supplementation, by supplying mitochondrial oxidative metabolism and energy production, was sufficient to promptly restore both muscle function and mass in tumor-bearing mice and in human subjects affected by cancer cachexia [9]. Hence, iron is essential for cellular metabolism and growth [10].

The importance of iron for life is principally due to its chemical properties, such as the ability to accept or donate electrons, interconverting between the ferrous (Fe^2+^) and ferric (Fe^3+^) states [11]. This capacity enables iron to bind oxygen and form complexes with many organic ligands [12]. However, its redox reactivity makes Fe^2+^ a potentially hazardous biometal involved in reactive oxygen species (ROS) generation through Fenton chemistry [13]. ROS, especially the highly aggressive hydroxyl radical, may damage proteins, DNA and lipids causing cellular toxicity and organ damage. In particular, the attack to membrane lipids in the process called lipid peroxidation promotes a specific type of regulated cell death called ferroptosis [14]. Given the toxicity of iron, particularly in excess, cells have developed several tight regulatory mechanisms to control the systemic and intracellular iron homeostasis, thereby maintaining adequate and safe amounts of iron [15].

## 2. Regulation of Cellular Iron Homeostasis

Cellular iron homeostasis in mammalian cells is regulated by balancing iron uptake, storage, export and utilization [16]. Cells mainly acquire iron from circulating iron-loaded holo-transferrin (Tf), which is internalized by clathrin-mediated endocytosis after binding to cell surface transferrin receptor 1 (TfR1) [17]. The binding affinity of Tf for Fe^3+^ is high at the physiological pH present at the cell surface and abolished at acidic pH. Therefore, the acidification of endosomes triggers the release of iron, which is then reduced to Fe^2+^ by the metalloreductase STEAP3 and transported across the endosomal membrane by DMT1 (divalent metal transporter 1) or other metal transporters like ZIP8 and ZIP14. The apoTf-TfR1 complex returns to the plasma membrane, where Tf dissociates from TfR1 [18]. The key role of TfR1 is shown by the severe defects caused by genetic inactivation of TfR1 (reviewed in [17]); however, other forms of uptake independent of the Tf–TfR1 interaction have been found, including receptor-mediated uptake of ferritin subunits (via TfR1, Scara5 and TIM-2), lipocalin 2 [15] and hyaluronan-CD44 complex [19]. The latter pathways have been mostly characterized in specific cell lines, but whether they have a general and functional role in iron uptake is still undetermined. Moreover, under conditions of iron overload, ZIP14 and ZIP8 may also mediate the uptake of circulating non-Tf-bound iron (NTBI) [20].

**Figure 1:**
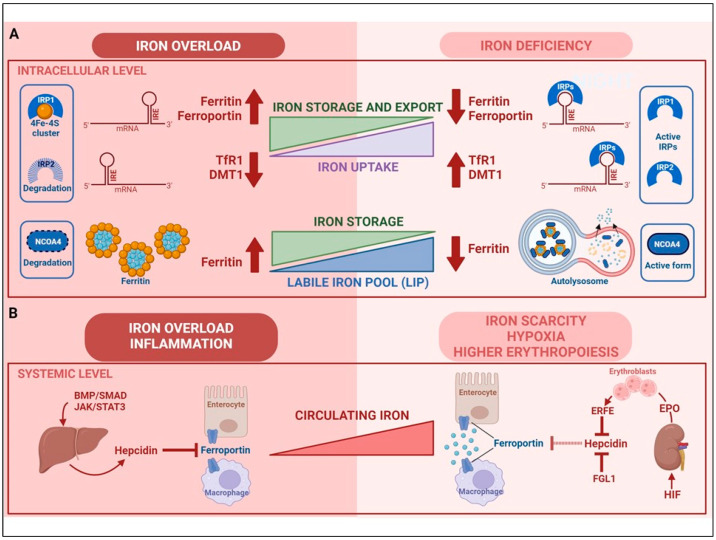
**Cellular and systemic iron metabolism**. (**A**) Simplified model of IRP-dependent and independent control of intracellular iron metabolism. Under conditions of iron deficiency, active IRP1 and IRP2 bind iron-responsive elements (IRE) at the 5′ untranslated region of ferritin and ferroportin mRNAs, thus preventing their translation, and to IREs in the 3′ end of TfR1 and DMT1 mRNAs, thus enhancing their stability and increasing iron uptake. These combined mechanisms lead to higher cellular iron availability. Conversely, excess iron facilitates the assembly of a 4Fe-4S cluster in IRP1, which loses its IRE binding activity and functions as cytoplasmic aconitase and triggers IRP2 proteasomal degradation. In the absence of active IRPs, ferroportin and ferritin are actively translated, so that superfluous iron is either stored or exported, whereas TfR1 and DMT1 mRNAs are degraded to prevent additional iron uptake. When cellular iron levels are low, NCOA4 “chaperones” ferritin shells to lysosomal destruction, thus increasing iron availability. Conversely, when iron is abundant, NCOA4 is targeted for degradation and iron is stored in ferritin. (**B**) Hepcidin-dependent regulation of body iron homeostasis. Hepcidin expression in the liver is induced by body iron stores and inflammatory signals via the BMP/SMAD and JAK/STAT3 pathways, respectively. Circulating hepcidin inhibits ferroportin-mediated iron export, primarily from reticuloendothelial macrophages and enterocytes, thus resulting in lower levels of iron in the bloodstream. Conversely, hepcidin synthesis is repressed by iron deficiency or when insufficient oxygen levels stimulate hypoxia inducible factor (HIF)-dependent erythropoietin (EPO) production in the kidney. EPO-stimulated erythroid precursors synthesize erythroferrone (ERFE), which in turn blocks BMP-mediated hepcidin transcription. This response allows for high ferroportin-mediated iron efflux into plasma from the gut and macrophages.

Once inside the cytoplasm, iron enters an incompletely characterized labile iron pool (LIP) where it loosely binds compounds like glutathione [21] and faces different fates: it may be stored in ferritin, utilized (e.g., for the synthesis of heme and iron-sulfur clusters) or released from the cell by ferroportin [22]. Iron export by ferroportin—described in detail below—is the major route of iron efflux; however, recent findings in experimental models indicated that secretion of iron-loaded ferritin in exosomes may represent an alternative pathway that may play a role in pathological conditions [15], such as liver fibrosis [23] and ferroptosis [24]. Recently, poly-(rC)-binding protein 1 (PCBP1) and PCBP2 were identified as cytosolic iron chaperones that are responsible for delivering Fe^2+^ to ferritin for oxidation and storage, and to ferroportin for export or for other non-heme iron requiring proteins [25]. Ferritin, a heteropolymer formed of 24 subunits of two types (H and L) [26], is able to accumulate up to 4000 iron atoms in a non-toxic (Fe^3+^) state and thus can have a dual function: iron storage and the prevention of oxidative damage [27]. In case of necessity, the iron deposited in ferritin shells can be released in a process involving NCOA4-mediated autophagic degradation of ferritin [20]. When iron levels are low, NCOA4 binds to ferritin and delivers it to the nascent autophagosome, which then fuses with the lysosome, thereby leading to ferritin degradation and iron release (Figure 1A). Given the thousands of iron atoms present in a ferritin shell, this process is expected to contribute substantially to cellular iron availability, as also demonstrated by studies showing the important role of NCOA4-dependent ferritinophagy in processes like erythropoiesis that requires large amounts of iron for hemoglobin synthesis (reviewed in [28]). When iron is abundant (at least in cell cultures), NCOA4 can also promote ferritin release in extracellular vesicles through a CD63-dependent pathway [29].

Cellular iron homeostasis is primarily regulated post-transcriptionally upon binding of iron regulatory proteins (IRP1 and IRP2) to iron-responsive elements (IREs) in the untranslated regions of the mRNAs for key proteins of iron metabolism [16] (Figure 1A). When intracellular iron levels are low, the active forms of IRP1 and IRP2 bind the IRE in the 5′ regions of ferritin and ferroportin mRNAs, thereby repressing their translation; at the same time, IRPs bind to the IREs at the 3′ of TfR1 and DMT1 mRNAs, protecting these transcripts from degradation and enhancing their expression [30]. By inhibiting iron storage and ferroportin-mediated iron export, while simultaneously favoring iron uptake, this response increases iron availability. Conversely, in iron-replete conditions, the inhibition of IRP activity reduces the uptake of unnecessary iron because TfR1 and DMT1 mRNAs are degraded, and allow the translation of ferroportin and ferritin mRNAs, thus favoring the storage and export of excess iron. Iron levels in the LIP modulate IRP activity by regulating the stability of IRP2, which in iron-replete conditions undergoes ubiquitination followed by proteasomal degradation, and controlling the switch of IRP1 between two different conformations. When iron is scarce, IRP1 is an apo-protein with IRE-binding activity, whereas, in the presence of sufficient iron, it is able to assemble a 4Fe-4S cluster, thus acquiring an enzymatic function as cytosolic aconitase [12] (Figure 1A). Notably, other proteins directly or indirectly linked to iron homeostasis are regulated by IRPs, including for example hypoxia inducible factor (HIF) 2α [31] (see below) and CD63 [29] (see above).

## 3. Regulation of Systemic Iron Homeostasis

The majority of the 3–5 g of iron contained in a healthy human body is present in red blood cells (RBC) as an essential component of hemoglobin and thus serves in oxygen transport. Significant amounts of iron are also present in the liver, in macrophages and in the myoglobin of muscles. Mammals do not possess any regulated mechanism for iron excretion from the body, as iron loss occurs from the sloughing of mucosal and skin cells or during occasional bleeding. Therefore, the balance is maintained by the tight control of dietary iron absorption. In duodenal enterocytes, dietary Fe^2+^ directly internalized or derived from heme destruction is exported across the basolateral membrane into the bloodstream by ferroportin. For the binding of iron to Tf, which delivers redox-inert Fe^3+^ to all body cells, the oxidation of Fe^2+^ is catalyzed by the membrane-bound ferroxidase hephaestin and its soluble homolog ceruloplasmin, which is necessary (of which more in “Mechanisms of iron absorption”).

However, it should be pointed out that Tf-bound iron, whose major role is to sustain erythropoiesis, is derived mostly by the iron recycling activity of reticuloendothelial macrophages, which clear effete RBC, destroy hemoglobin and heme, and export iron via ferroportin into the bloodstream where the metal is oxidized by ceruloplasmin and loaded to Tf [32].

Systemic iron balance is maintained by the hepcidin–ferroportin axis, which coordinates absorption, recycling, utilization and storage [33] (Figure 1B). Hepcidin, a liver-derived peptide hormone, binds to ferroportin and thus induces its internalization and degradation, thereby blocking iron efflux (mainly from enterocytes and macrophages) [34]. In a negative-feedback process, elevated hepcidin transcription, which is mainly induced by high iron availability through the bone morphogenic proteins (BMP)-SMAD1/5/8 pathway and inflammatory mediators like IL-6 through the JAK-STAT3 pathway, leads to a decrease in circulating iron levels [35]. Conversely, under conditions of enhanced erythropoiesis, hepcidin expression is inhibited mainly by erythroferrone (ERFE), which interferes with the BMP pathway, thus leading to higher ferroportin-mediated iron release into plasma in order to meet the increased iron demand for RBC synthesis [36] (Figure 1B). Ultimately, the latter pathway is controlled by the drop in oxygen levels associated with the higher erythropoietic drive. In fact, ERFE expression in erythroid precursors is induced by erythropoietin [37], which in turn is synthesized by kidney cells in response to the enhanced activity of HIF, a transcription factor activated by both hypoxia and low levels of iron [38]. Accordingly, it has been recently shown that low oxygen levels in the liver induce the synthesis of another hepcidin inhibitor, the fractalkine FGL1, which inhibits hepcidin by binding to BMP6 [39] (Figure 1B). Tough iron, inflammation and erythropoiesis are the major regulators of hepatic hepcidin expression, it has been shown that other conditions, e.g., endoplasmic reticulum stress [40], hormones, e.g., leptin [41] or metabolites (e.g., lactate) [42] may affect hepcidin-mediated control of iron homeostasis. Moreover, smaller amounts of hepcidin produced in organs like the lung, the heart, and the intestine, can modulate iron metabolism locally [33].

## 4. Iron Absorption

Multicellular organisms evolved by recycling nutrients like iron. However, our body, despite an amount of recycled iron around 25 times greater than the quantity acquired from the diet, needs to assimilate iron from food on a regular basis. In theory, given that a balanced diet contains 10–30 mg of iron, the amount of 2 mg iron per day necessary to compensate for non-specific iron losses should be easily obtained [43]. However, under the oxidative conditions characterizing the biosphere, environmental iron is insoluble; thus, the paradoxical contradiction between the great abundance of iron and its poor bioavailability extends to dietary iron, which indeed is mostly inorganic iron found in food derived from both plants and animals. This form of iron is absorbed with an efficiency of around 10% depending on the type of diet, with a range between 14 and 18% of iron absorbed from mixed diets and 5 and 12% from vegetarian diets. This difference is explained by the greater absorption efficiency of heme iron, primarily present in hemoglobin and myoglobin from animal food sources, which is around ~25% [44]. Indeed, though heme consists of ~10–15% of total dietary iron sources in meat-eating populations, it accounts for over 40% of assimilated iron [45]. The reason for the better absorption of heme is not clear and may depend on its lipophilic properties. However, the latter explanation is not in line with evidence that not all mammals show this preference for heme as their major iron source; in fact, for example, mice absorb dietary heme poorly [46], thereby making this useful animal model inadequate for the characterization of this still poorly understood mechanism (see below).

It should be noted that the small fraction of iron absorbed under physiological conditions may increase substantially if body iron stores are depleted or the iron demand is increased; in an iron-deficient individual a maximum absorption at 20% and 35% for inorganic and heme iron, respectively, has been reported [45]. Similarly, nutritional guidelines recommendations take into account the higher requirements of infants, menstruating and pregnant women, who should obtain 2–3 times more daily iron from their diet than the 10 mg value appropriate for adult men.

### Mechanisms of Iron Absorption

**Figure 2:**
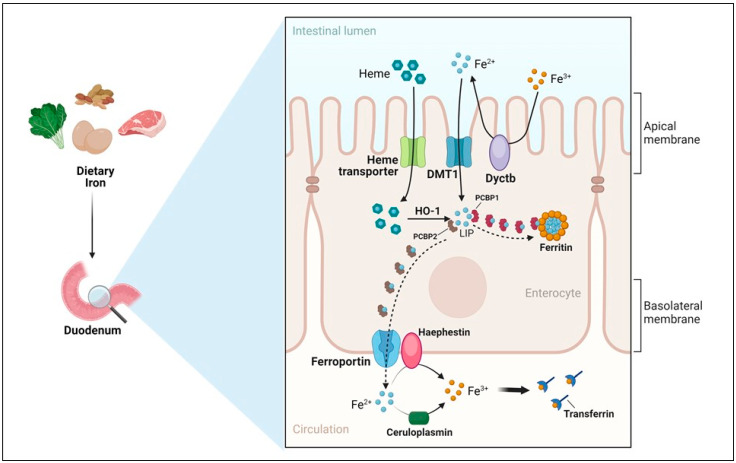
**Dietary iron absorption.** Dietary iron is mainly composed of poorly absorbed inorganic iron, which is found in food derived from both plants and animals and is mostly in the ferric (Fe^3+^) form. Heme iron, primarily present in animal food sources like meat, though less abundant is more bioavailable. The first step of duodenal iron absorption is the transport of ferrous (Fe^2+^) iron by DMT1 at the apical surface of enterocytes. Previous reduction of the predominant Fe^3+^ by Dcytb is necessary. Iron bound to heme is internalized by a still unknown importer and iron is then released in the cytoplasm by the degradative action of heme oxygenase (HO-1). Following the brush border transit, both the iron imported by DMT1 and that liberated from heme enter the labile iron pool (LIP) and are either utilized, incorporated in ferritin shells or exported by ferroportin. PCBP1 and PCBP2 function as chaperones that deliver iron to client proteins. At the basolateral surface, following the efflux of Fe^2+^ into the bloodstream by ferroportin, the oxidative action of hephaestin and ceruloplasmin is required for the binding of Fe^3+^ to circulating transferrin.

The transit of iron from the duodenal lumen to the bloodstream can be divided into three major steps: (a) *apical uptake* (the transport across the brush border), (b) *enterocytic intracellular phase* (iron utilization, storage or export) and (c) *basolateral transfer* (the release from the enterocytes to the circulation) (Figure 2). The relative importance of these stages, which are controlled by pathophysiological signals has been long debated, but recent findings, including the characterization of the hepcidin/ferroportin axis, seem to indicate that the basolateral transfer is more critical, as first suggested by studies showing that high release of iron from the intestine into the bloodstream underlies the inappropriate iron absorption found in hemochromatosis patients [47].

(a) *Apical uptake.* The uptake of nutritional nonheme iron, which has been elucidated at the molecular level, occurs mostly in the first portion of the duodenum and involves the transport of Fe^2+^ across the apical membrane of enterocytes by DMT1 (Figure 2). However, since Fe^3+^ is the form of iron mostly present in the diet and the low pH present in the intestinal lumen is not sufficient to maintain iron in a soluble form, the previous reduction of iron by ferric reductases, such as Dcytb (duodenal cytochrome *b*) is required [15]. Accordingly, the importance of diet composition in determining the amount of iron absorbed is well recognized. Indeed, the assumption of reductants like vitamin C can improve iron absorption by making the task of Dcytb easier. Conversely, food components mainly present in vegetables like phytates, which are primarily found in cereals and legumes, or tannins, may reduce iron absorption because of unspecific binding, physical entrapment and decreased intestinal transit time. The strategy aimed at achieving better iron bioavailability by decreasing the consumption of food containing these inhibitors should be matched against the recent and warranted trend toward increasing the intake of insoluble fiber. However, phytases to remove phytic acid from food are increasingly used in food-processing techniques to reduce these inhibiting effects. Other nutrients, including minerals like calcium and vitamins, could possibly impair iron absorption (reviewed in [48]). Dietary heme, originating primarily from meat and seafood, can also be transported across the apical membrane by a hitherto poorly known mechanism. In fact, heme carrier protein 1 (HCP1), which was initially identified as an intestinal heme importer, turned out to transport folate, for which HCP1 has an affinity much higher than for heme. Alternatively, heme responsive gene (HRG1) that transports heme across the erythrophagosomal membranes of macrophages during iron recycling from RBC [49] and is expressed in the human small intestine, could represent a candidate for intestinal heme absorption, but its role in this context is still unknown [44]. In any case, it is well established that dietary absorbed heme is subsequently catabolized within intestinal epithelial cells by heme oxygenase 1 (HO-1) to liberate Fe^2+^, which then follows the same destiny of inorganic iron imported by DMT1.
(b) *Enterocytic intracellular phase.* Internalized Fe^2+^ enters the LIP in the enterocytic cytoplasm and, as in any other cell, is either utilized, incorporated in ferritin, or exported by ferroportin at the basolateral surface (see below) (Figure 2). Given the function of the duodenum in body iron absorption, the latter fate is predominant. Recently, a key role for the chaperone PCBP1 in intestinal iron absorption has been reported [50]; the cell-specific deletion of PCBP1 in mice led to lower iron and ferritin levels in enterocytes and disrupted iron balance. As already mentioned, iron not used by the duodenal cells is either reversibly stored in ferritin or exported by ferroportin. Whether ferritin levels simply reflect the iron status of the enterocyte or play an active role in the control of absorption has been long discussed. Indeed, we found that in line with the corresponding IRP binding activity, ferritin expression in duodenal biopsies was higher than normal in patients with iron overload and lower in iron-deficient patients with the exception of the inappropriately low levels found in patients with genetic hemochromatosis which is characterized by inappropriately high iron absorption [51,52]. Conversely, the cell-specific deletion of H ferritin in duodenal cells leads to unrestrained absorption and body iron overload [53], whereas ferritin overexpression caused by IRP inactivation has the opposite effect [54]. The current view is that IRP-mediated cell-autonomous regulation of ferritin synthesis sets a basal level of ferritin, which represents a temporary sink for iron not transferred to the circulation and is then lost when the apical cells are sloughed. These new findings provided a novel view of the mucosal block model proposed decades ago [55], but other control mechanisms, in particular, ferroportin-mediated basolateral transfer and the discovery of NCOA4-mediated ferritinophagy add complexity to this pathway. NCOA4 is required to avoid iron trapping in enterocytes when the demand for iron is high; however, a recent study showed quite surprisingly that in mice with intestine-specific deletions of NCOA4 iron homeostasis is not altered under normal conditions or in iron deficiency. In these settings, NCOA4 may be regulated by the HERC2 E3 ubiquitin-protein ligase, which triggers its proteasomal degradation [56]. Conversely, in a mouse model of genetic iron overload, the silencing of NCOA4 in enterocytes favored iron retention in the duodenum and mitigated systemic iron loading [57]. These findings, which are in line with the inappropriately low expression of both H and L ferritin subunits previously detected in the duodenal biopsies of patients with genetic hemochromatosis [52], suggest that the local inhibition of NCOA4 activity with consequent iron trapping within enterocytic ferritin may represent a novel therapeutic approach to limit iron uptake in the clinical conditions characterized by iron hyperabsorption.
(c) *Basolateral transfer.* As anticipated above, the final step of intestinal iron absorption is represented by the efflux of Fe^2+^ at the basolateral surface, which is accomplished through conformational changes in ferroportin [58] (Figure 2). The key role of ferroportin in dietary iron absorption was shown by the rapid insurgence of anemia in adult mice in which ferroportin was specifically deleted in intestinal cells [59]. Eventually, the combined effect of two multicopper ferroxidases, membrane-bound haephestin and circulating ceruloplasmin, facilitates iron efflux and allows for Fe^3+^ loading onto plasma Tf for distribution [45,60]. The characterization of their role in iron absorption and mobilization provided a molecular basis for the findings of earlier elegant studies showing that a copper-deficient diet leads to iron deficiency anemia in pigs [61]. Given that strong variations in Tf saturation do not affect iron absorption in both mouse models and patients, Tf was thought to be only a passive iron acceptor. However, a recent study showing that lamina propria macrophages, in response to inflammation and iron, can produce proteases that degrade Tf locally in the interstitium, thus impairing ferroportin-dependent iron export [62], suggests that Tf may play an unexpected role in body iron absorption.

Interestingly, mutant mice lines were instrumental in identifying, cloning and characterizing the genes involved in both apical and basolateral stages of iron absorption. In fact, the severe anemia of the microcytic (mk) mouse and the Belgrade (*b*) rat is due to identical missense mutations in the DMT1 gene [63,64]. Similarly, McKie and colleagues identified Dcytb [65] and ferroportin [66] in the duodenum of hypotransferrinemic (hpx) mice, while positional cloning of the gene defective in the sex-linked anemia (sla) mouse resulted in the identification of hephaestin [67]. The severe phenotypes of all these mutant mice underscore the key role of the corresponding genes in iron metabolism.

## 5. Regulation of Iron Absorption

Both local and systemic pathways control duodenal iron absorption. In fact, the three major players in these settings appear to be the hepcidin/ferroportin axis, the IRE/IRP regulatory network and the oxygen-iron regulated HIF system. This complex regulation provides an efficient system to maintain a response to iron requirements that is both fast (HIF-dependent transcriptional control) and sustained (long-term responses most likely mediated by the hepcidin/ferroportin axis) [68]. The first two pathways have been described above. Regarding the third, it is important to underline that both hypoxia and iron deficiency activate the HIF response. In fact, the prolyl hydroxylases (PHD) that catalyze prolyl hydroxylation of HIF, thereby targeting it to ubiquitination and proteasomal degradation, require oxygen and 2-oxoglutarate as substrates, but also iron as a cofactor [38]. Notably, though PHD regulates both HIF1α and HIF2α, only the latter is crucial for iron absorption [69].

These three pathways interact in a complex interplay, as IRPs regulate the translation of HIF2α mRNA [31], whereas several genes coding for the proteins of iron metabolism are transcriptional targets of HIF. In the context of duodenal cells, particularly important targets are DMT1, Dcytb, ferroportin and NCOA4 [70].

Under conditions of low hepcidin levels when iron uptake should be maximized, ferroportin-mediated iron export leads to a contraction of the LIP and consequent IRP activation in duodenal enterocytes. This results in higher DMT1 and Dcytb import activity on the apical membrane and repression of ferritin synthesis, thereby limiting iron storage. Ferroportin mRNA escapes the negative regulation by IRP because a variant transcript lacking the IRE is expressed in the duodenum [71]. At the same time, iron scarcity caused by ferroportin-mediated iron efflux prevents PHD activity and stabilizes HIF2α, which induces the transcription of DMT1, Dcytb, NCOA4 and ferroportin, thus activating apical uptake, preventing the accumulation of ferritin and enhancing export. The elucidation of the crosstalk between the liver and the intestine that is mediated by the hepcidin–HIF2α axis [72] provides insights into the pathways upregulating iron uptake in response to body iron deficiency as well as excess iron absorption under pathological conditions characterized by hepcidin deficiency.

On the contrary, under conditions of iron excess or inflammation, the downregulation of ferroportin by hepcidin raises enterocyte iron levels, which in turn increases PHD-dependent degradation of HIF and inactivates IRPs. These mechanisms result in the down-regulation of all the proteins involved in iron absorption indicated above at the transcriptional and post-transcriptional levels.

As previously mentioned, a recent study demonstrated that PCBP1 plays an important role in this context, although the molecular mechanisms remain to be fully elucidated [50].

## 6. Examples of Diseases Related to Iron Absorption

As stated above, insufficient iron uptake can lead to anemia or nonanemic iron deficiency, which are among the most common diseases worldwide [73]. Often, this occurs when the physiological needs for iron are increased at certain times in life (e.g., growth, pregnancy). In some cases, the underlying cause is genetic, like iron refractory iron deficiency anemia (IRIDA), a rare recessive disorder characterized by hypochromic microcytic anemia and very low transferrin saturation. IRIDA patients bear mutations within the TMPRSS6 gene, which encodes matriptase-2, a transmembrane serine protease that negatively regulates hepcidin [74]. The resultant high hepcidin levels impair intestinal iron absorption and iron recycling by macrophages, thus making therapy very difficult, as oral iron is not absorbed and most intravenous (IV) iron is trapped within the reticuloendothelial system [75]. Interestingly, recent, though still limited, evidence suggests that polymorphisms in the TMPRSS6 gene may have a more general role in iron deficiency and anemia [76] (Table 1). However, the most frequent causes of iron deficiency anemia (IDA) are iron-poor diets, in particular, those lacking animal-source foods that contain heme iron. In these cases, iron absorption is not intrinsically defective and may be even increased (Table 1). Iron deficiency is found also in more than 50% of patients with celiac disease (CD), a chronic immune-mediated pathology affecting the small intestine triggered by exposure to dietary gluten in genetically predisposed individuals [77]. In these patients, the proximal duodenum where iron absorption occurs is typically damaged; therefore, only IV iron therapy can be considered for patients with active CD, whereas oral iron can be used in patients in which gluten-free diet ameliorated intestinal atrophy. Notably, in some patients, the normalization of alterations of the intestinal mucosa is sufficient to recover from iron deficiency and anemia without iron supplementation.

Anemia can also be present in patients with chronic inflammation (ACD) accompanied by sustained hepcidin production [78] (Table 1). However, in chronic inflammatory bowel diseases (IBD) like Crohn’s disease, intestine-specific defects, by impairing the absorptive capacity of the gut, may also hamper to some extent iron uptake, though the associated anemia may be mainly caused by malabsorption of vitamin B12/folate, blood loss and the concomitant inflammation [79]. In this regard, it can be speculated that the iron-deficient anemia affecting patients with another IBD, such as ulcerative colitis (UC) is likely due to the associated inflammation, as iron is not absorbed in the distal intestine that is affected in UC. In these settings, an adequate therapeutic response is obtained with IV iron administration that bypasses the gastrointestinal tract.

On the other hand, there are several pathological conditions that directly or indirectly cause hyperabsorption, thus leading to iron overload (Table 1). The most common is HH, a group of genetic disorders in which hepcidin deficiency leads to systemic iron excess [80]. In most cases, hemochromatosis is caused by homozygous mutations in the gene encoding HFE which has a role in hepcidin regulation. Hepcidin production is impaired and inappropriately responding to iron, but still detectable; therefore, the penetrance of the disease is low. Loss-of-function mutations in other genes encoding hepcidin (HAMP), hemojuvelin (HJV) and transferrin receptor 2 (TFR2) or gain-of-function mutations in the gene encoding ferroportin (SLC40A1) cause much rarer forms of hemochromatosis (non-HFE HH), but in general, iron accumulation proceeds at the higher rate, often leading to juvenile forms of the disease. In all the forms, the pathophysiological mechanism is unregulated iron absorption due to defects in the hepcidin/ferroportin axis.

Other widespread genetic diseases affecting iron absorption are hemoglobinopathies (Table 1). In thalassemia, the imbalance in the production of α and β-globin chains due to mutations in the α- and β-globin gene clusters (HBA1 and HBB) results in ineffective erythropoiesis, chronic hemolytic anemia and compensatory hemopoietic expansion [81]. In non-transfusion-dependent thalassemic patients who maintain acceptable hemoglobin values, despite the presence of excess iron, hypoxia-induced and ERFE-mediated hepcidin suppression chronically stimulates iron absorption, eventually resulting in massive iron overload. Patients with more severe forms of thalassemia, which require chronic RBC transfusions to survive, develop very severe secondary iron overload and iron chelation therapy is necessary to prevent organ damage. In sickle cell disease (SCD), the premature breakdown of RBC caused by a single amino acid substitution in β-globin ultimately results in hemolytic anemia. Chronic hemolysis enhances iron demand for erythropoiesis, thereby prompting intestinal hyperabsorption of iron; however, iron overload occurs only with blood transfusion [82]. In fact, in contrast to thalassemia where ineffective erythropoiesis leads to impaired production and higher destruction of RBC in the bone marrow, in SCD intravascular hemolysis provides a potential mechanism for iron elimination through increased urinary loss and biliary excretion. Paradoxically, dietary iron restriction ameliorates anemia and other clinical consequences of SCD [83], possibly by affecting the microbiota, at least in mouse models [84].

While the pathological consequences of elevated iron stores due to excessive dietary iron intake have been conclusively demonstrated for conditions like hereditary hemochromatosis, which is characterized by an iron absorption rate inappropriate to iron stores [85], the evidence from epidemiological studies that high dietary iron intake may predispose to diseases is still not fully convincing [86], and may be jeopardized by the confounding factors associated with the studies investigating food consumption. However, since high body iron stores have been associated with pathologies highly relevant to human health like cancer, metabolic syndrome and cardiovascular diseases [43], further investigation should be performed.

Iron that is not absorbed in the duodenum eventually arrives at the distal sections of the intestine where it can fuel microbial growth and possibly influence the intestinal microbiota, which has been shown to play a relevant role in a broad range of metabolic and physiologic processes [87]. Since different bacteria have distinct iron requirements for growth and survival, iron availability can play a role in altering the microbial profile in the gut. In particular, excess iron can favor potentially pathogenic enterobacteria species at the expense of protective lactobacilli and bifidobacteria that need little iron [86]. Consequently, any up-to-date iron therapy needs to consider the effects on the microbiota (see below).

Interestingly, it has also been shown that metabolites produced by various bacterial species can inhibit duodenal absorption by repressing HIF-mediated expression of iron transporters and inducing ferritin [88]. Using this strategy, aimed at “stealing” iron from the host, gut microbes obtain more iron for their own necessities.

## 7. Therapy

Various approaches have been considered to address the problem of insufficient iron availability, which remains the main public health issue related to iron absorption. Interventions include food processing, as described above for the use of phytase, food fortification and supplementation [48]. Fortification, i.e., the addition of iron to processed foods, represents a successful strategy, which, however, requires careful evaluation of technological, commercial, nutritional and social issues. In general, appropriately designed and implemented fortification strategies were able to ensure a sufficient iron intake to prevent the pathological consequences of iron deficiency, primarily anemia [48].

On an individual basis, direct oral supplementation is the most common therapy involving intestinal absorption, as IV therapy, which remains the therapy of choice for rapid corrections or for specific types of patients, obviously bypasses the gut route. Iron preparations usually rely on Fe^2+^ because it has higher bioavailability than Fe^3+^, though the most commonly used forms, such as ferrous sulfate, sometimes cause gastrointestinal side effects that may lead to reduced compliance with therapy. Since the usual doses of oral iron trigger an increase in hepcidin, accompanied by a reduction in iron absorption, lasting at least 24 h, alternate day dosing may represent a way to maximize absorption [86]. Recently, new oral iron therapies based on Fe^3+^ have been developed. The maltol–iron complex proved to be both tolerable and effective [89]. In fact, by making iron stable, iron bioavailability is increased and the risk of both mucosal toxicity and modifications of the gut microbiota is reduced. Moreover, high iron bioavailability and excellent gastrointestinal tolerance characterize sucrosomial iron, an oral iron formulation in which Fe^3+^, protected by a phospholipid bilayer and a sucrester matrix, is absorbed through para-cellular and trans-cellular routes [90].

An alternative strategy to improve iron status without iron supplementation could be to improve the efficiency of absorption by exploiting the transmembrane iron gradient between the gut lumen and the enterocytes. To this regard, hinokitiol, a small molecule natural product isolated from plants that strongly binds both Fe^2+^ and Fe^3+^ was shown to increase gut iron absorption in two experimental models with impaired apical and basolateral transfer, the Belgrade (b) rats (with a loss of function mutation of DMT1) and ferroportin deficient flatiron mice, respectively [91].

As reported above, since eukaryotic cells and microbes compete for iron, nutritional immunity, i.e., the restriction of available iron by the host [92] is a defense strategy from infection so important that is widely diffused and is present also in plants. Indeed, it has been shown that when bacteria threatening plant health enter root tissues, iron acquisition from the soil is inhibited through the degradation of the iron-deficiency signaling peptide Iron Man 1 [93]. Therefore, the need to eliminate inadequate iron supply without exceeding upper tolerable intakes stems from the ample evidence that iron administration can increase the risk of several infections. To summarize a complex and evolving field, recent evidence suggests that excessive doses of supplemental iron can indeed increase the risk of bacterial and protozoal infections (particularly malaria) by excessively enhancing Tf saturation and thus leading to the formation of NTBI [86]. However, less aggressive interventions, based for example on fortified food and providing lower amounts of iron, appear generally safer and represent the best balance of risk and benefits. Moreover, as discussed above, unabsorbed iron may have a negative impact on health by leading to microbiota dysbiosis. Thus, accurate dosing should be adopted. In these settings, modern stable iron isotope techniques are a promising new method to accurately quantify iron absorption and assess the impact of interventions [94].

## 8. Conclusions

Thanks to the identification of critical molecules involved in iron transport, our view of intestinal iron absorption is now more complete and more detailed. However, this highly regulated process is far from being completely unraveled. For example, the transport of heme, which is the most important source of dietary iron, still awaits the identification of the apical transporter.

Traditionally, iron absorption was thought to be regulated by body iron requirements, with iron stores and erythropoietic activity being the two main players [95]. More recently, inflammation, which stimulates hepcidin synthesis, and the microbiota, which mutually interacts with absorptive duodenal cells, have been shown to be other important factors.

The identification of the key proteins involved in intestinal iron absorption and the increased knowledge about the control of their expression and activity has an important translational aspect related to iron supplementation, a key global health issue in the context of the intensive global efforts to combat iron deficiency and the associated anemia.

Available evidence indicates that iron given as supplements in non-physiological amounts can increase the risk of microbial infections; moreover, the availability of unabsorbed iron to the microbial communities of the gut may be dangerous and lead to dysbiosis.

However, lower quantities of iron within a matrix or in highly bioavailable formulations are probably less dangerous, thereby representing an approach that offers the best balance of risk and advantages. Therefore, also in this context, the yin-yang dual potential of iron applies and should be considered in public health programs and recommendations.

## Figures and Tables

**Table 1:** **Major genetic and acquired iron absorption-related disorders**. The table summarizes a list of major genetic and non-genetic iron absorption-related disorders (indicated in bold). Genetic and non-genetic causes are reported in table. For genetic diseases the gene target of mutation and the biological function of associated protein are reported. Final alterations of iron absorption with relative underlying molecular mechanisms are shown.

Disease	Non Genetic Cause	Mutated Gene	Function of Altered Target Protein	Molecular Basis of Altered Iron Absorption	Iron Absorption
**IRIDA**	-	TMPRSS6	Negative regulation of hepcidin	Hepcidin increase	Reduced ↓
**IDA**	Diet (major cause)	-	-	Hepcidin decrease	Increased ↑
**ACD**	Inflammation	-	-	Hepcidin increase	Reduced ↓
**HH**	-	HFE	Modulation of hepcidin production	Inappropriate hepcidin decrease	Increased ↑
**Non-HFE HH**	-	HAMP	Down-regulation of ferroportin	Loss of hepcidin regulation	Increased ↑
		TFR2 HJV	Regulation of hepcidin expression		
		SLC40A1	Iron export	Hepcidin resistance	
**Thalassemia**	-	HBA1 HBB	Hb formation	ERFE-mediated hepcidin repression	Increased ↑
***SCD***	-	HBB	Hb formation	ERFE-mediated hepcidin repression (to be confirmed)	Increased ↑

## Data Availability

Not Applicable.
